# Triple-Negative Breast Cancer Analysis Based on Metabolic Gene Classification and Immunotherapy

**DOI:** 10.3389/fpubh.2022.902378

**Published:** 2022-07-06

**Authors:** Yu Zhou, Yingqi Che, Zhongze Fu, Henan Zhang, Huiyu Wu

**Affiliations:** ^1^Oncology Department, The First Affiliated Hospital of Jiamusi University, Jiamusi, China; ^2^Hematology-Oncology Department, Long Nan Hospital, Daqing, China; ^3^Gastroenterology Department, The First Hospital of Qiqihar, Qiqihar, China; ^4^Third Department of Oncology, People's Hospital of Daqing, Daqing, China

**Keywords:** triple-negative breast cancer, metabolic genes, bioinformatics, molecular typing, tumor microenvironment, immunotherapy

## Abstract

Triple negative breast cancer (TNBC) has negative expression of ER, PR and HER-2. TNBC shows high histological grade and positive rate of lymph node metastasis, easy recurrence and distant metastasis. Molecular typing based on metabolic genes can reflect deeper characteristics of breast cancer and provide support for prognostic evaluation and individualized treatment. Metabolic subtypes of TNBC samples based on metabolic genes were determined by consensus clustering. CIBERSORT method was applied to evaluate the score distribution and differential expression of 22 immune cells in the TNBC samples. Linear discriminant analysis (LDA) established a subtype classification feature index. Kaplan-Meier (KM) and receiver operating characteristic (ROC) curves were generated to validate the performance of prognostic metabolic subtypes in different datasets. Finally, we used weighted correlation network analysis (WGCNA) to cluster the TCGA expression profile dataset and screen the co-expression modules of metabolic genes. Consensus clustering of the TCGA cohort/dataset obtained three metabolic subtypes (MC1, MC2, and MC3). The ROC analysis showed a high prognostic performance of the three clusters in different datasets. Specifically, MC1 had the optimal prognosis, MC3 had a poor prognosis, and the three metabolic subtypes had different prognosis. Consistently, the immune characteristic index established based on metabolic subtypes demonstrated that compared with the other two subtypes, MC1 had a higher IFNγ score, T cell lytic activity and lower angiogenesis score, T cell dysfunction and rejection score. TIDE analysis showed that MC1 patients were more likely to benefit from immunotherapy. MC1 patients were more sensitive to immune checkpoint inhibitors and traditional chemotherapy drugs Cisplatin, Paclitaxel, Embelin, and Sorafenib. Multiclass AUC based on RNASeq and GSE datasets were 0.85 and 0.85, respectively. Finally, based on co-expression network analysis, we screened 7 potential gene markers related to metabolic characteristic index, of which CLCA2, REEP6, SPDEF, and CRAT can be used to indicate breast cancer prognosis. Molecular classification related to TNBC metabolism was of great significance for comprehensive understanding of the molecular pathological characteristics of TNBC, contributing to the exploration of reliable markers for early diagnosis of TNBC and predicting metastasis and recurrence, improvement of the TNBC staging system, guiding individualized treatment.

## Introduction

Breast cancer is the most common malignant tumor in women, accounting for about 25% of female malignant tumors (1) and also a leading cause of cancer deaths in women worldwide, with a significant increase in recent years (2). According to immunohistochemical characteristics, breast cancer is clinically classified into four types including luminal A type, luminal B type, human epidermal growth factor receptor 2 (HER2) overexpression type, and triple-negative breast cancer (TNBC). Among them, TNBC refers to a special type of breast cancer with negative expression of estrogen receptor, progesterone receptor and human epidermal growth factor receptor 2, which is characterized by high mitotic rate, easy lymphocyte infiltration, high degree of malignancy and larger tumor size and other characteristics (3). TNBC does not have effective endocrine therapy and targeted therapy. The currently effective systemic treatment is mainly chemotherapy. Some patients often develop recurrence or distant metastasis within a short period of time after chemotherapy, and the prognosis is poor (4). Thus, TNBC is clinically called “refractory breast cancer”.

The occurrence, development and metastasis of breast cancer are caused by the interaction between tumor cells and the microenvironment of the tumor. It involves not only tumor suppressor or oncogene mutations, but also tumor cells themselves, immune cells, extracellular matrix components, and tumor renewal supporting blood vessels and other components jointly suppressing the changes in the immune microenvironment (5). Previous studies have shown that the TME can affect the gene expression of tumor tissues in many ways, thereby affecting the occurrence and development of tumors (6). For example, by using the negative regulatory mechanism of the immune system, tumor cells can regulate the TME. A full range of immunosuppressive states can be used to counter the body's antitumor immunity (7). Individual differences in the efficacy of tumor immunotherapy are closely related to immunosuppression in the TME (8). Stromal cells and immune cells infiltrating tumor tissues constitute the main components of the dynamic network of the TME. TNBC is the subtype of breast cancer most closely related to the tumor immune microenvironment. Its high genetic instability and mutation burden could lead to neoantigens. It can be easily recognized by the immune system, making it one of the tumor types suitable for receiving immunotherapy intervention (9).

Currently, clinical pathological staging is commonly used to assess the prognosis of breast cancer patients. However, triple-negative breast cancer is highly heterogeneous, which affects the effectiveness of routine prognostic evaluation. In recent years, more and more studies have focused on the exploration of the biological characteristics of TNBC and the evaluation of prognostic factors. Among them, AR, p53, CK5/6, EGFR, and Ki-67 are more commonly used pathological indicators (10, 11). Study has reported the important role of these indicators alone or in combination in the progression of breast cancer (12). Although the overall prognosis of TNBC is poor and it is prone to visceral and central nervous system metastasis, personalized treatment for different subtypes of TNBC, such as the treatment of androgen receptor-positive androgen receptors, may have a better therapeutic effect. Body inhibitors (13), immune checkpoint inhibitors for the treatment of immune-related subtypes (9, 14), EGFR inhibitors based on gene expression/amplification (15, 16), and PI3K/AKT/mTOR signals targeted therapy drugs such as pathways (17, 18) will provide a reliable theoretical basis for the “precision treatment” and prognosis evaluation of TNBC. At present, the clinical further molecular classification of TNBC has not yet been carried out, and the existing classification is still in exploratory stage without general consensus. Therefore, how to type TNBC at the molecular level and better guide the treatment of TNBC still needs more in-depth research.

With the development of gene chip and high-throughput sequencing technology, based on the big data of the GEO database and TCGA database, comprehensive and systematic analysis of tumor-related genes and their regulatory mechanisms using bioinformatics methods is an important part of the current tumor genomics study. Research methods (19). Metabolic disorders, as one of the essential characteristics of tumors (20), affect a variety of tumor biological behaviors including occurrence, development, metastasis and recurrence (21). On one hand, carcinogenic factors disrupt metabolic balance, induce metabolic reorganization, and cell carcinogenesis, on the other hand, the reorganized metabolic system mediates a variety of biological behaviors and participates in the proliferation, invasion and metastasis of cancer cells (22). Therefore, molecular typing based on metabolism of triple-negative breast cancer is useful for a comprehensive understanding of the molecular pathological characteristics of triple-negative breast cancer, exploration of reliable markers for early diagnosis and prediction of metastasis and recurrence of triple-negative breast cancer as well as for perfecting the TNBC staging system. Individualized treatment is of great significance to improve the diagnosis and treatment of TNBC.

For this purpose, in this study, we divided TNBC into different metabolic molecular subtypes based on metabolism-related genes, and compared the molecular pathological characteristics of different metabolic subtypes of TNBC in multiple dimensions. The immune characteristics of different metabolic molecular subtypes and their different response modes to immunotherapy were analyzed. At the same time, the correlation between immune checkpoints, grouping of different metabolic molecular subtypes, and the differences in molecular mutations were compared. Finally, potential prognostic markers related to the metabolic characteristic index were screened. In short, we established a TNBC molecular typing model based on metabolic characteristics, and developed the immune characteristic index of each subtype based on this, so as to supplement the current clinical staging system. Our research results will provide research ideas and a theoretical basis for prognostic estimation and individualized treatment of OC patients.

## Materials and Methods

### Expression Profile Data and Metabolism-Related Genes

We used TCGA GDC API to download TCGA-BRCA RNA-Seq data and clinical survival and characteristic information. The (METABRIC, Nature 2012 & Nat Commun 2016) dataset (METABRIC dataset) (23) with TNBC sequencing data from the cbioportal website (https://www.cbioportal.org/) was acquired. We downloaded the GSE103091 (24) and GSE31448 (25) chip datasets with survival time from the Gene Expression Omnibus (GEO) database on April 07, 2021. We collected metabolic-related gene sets from previous studies (26) after sorting out a total of 2,752 genes.

### Data Preprocessing

TCGA-BRCA's RNA-Seq data processing: (1) Samples without clinical follow-up information, survival time, or survival status were removed; (2) Ensembl ID was converted to gene symbol according to the gene transfer format (GTF) file (Release 40) downloaded from GENCODE (https://www.gencodegenes.org/human/); (3) The median expression values were selected for multiple gene symbols of one gene; (4) Filtering the genes whose expression in the sample was <0.5 and account for more than 50%; (5) Keep samples of triple-negative breast cancer; METABRIC dataset processing: (1) Remove normal samples; (2) Remove the samples without survival time and survival status. Then we combined the expression profiles of TCGA and METABRIC datasets by using the removeBatchEffect function in the limma package (27) to remove batch effects between different datasets (referred to as RNASeq datasets). The combined expression profiles named RNASeq cohort in the following. Finally, a total of 414 samples with 235 alive and 179 dead samples remained in the RNASeq cohort.

GSE dataset processing: (1) Remove samples without clinical follow-up information, survival time or survival status; (2) Keep triple-negative breast cancer samples; (3) Convert probes to gene symbol; (4) Remove the probe when corresponding to multiple genes; (5) The median expression value was selected when there were multiple gene symbols of one gene; (6) Combine GSE103091 and GSE31448, and use the removeBatchEffect function of the limma package (27) to remove batch effects between different datasets (referred to as GSE dataset). Finally, a total of 188 samples with 134 alive and 54 dead samples remained in the GSE dataset.

### Subtype Classification of TNBC

Single-factor analysis was used to screen prognostic-related “metabolic genes”. Through consensus clustering (ConsensusClusterPlus) (28), 414 TNBC samples were clustered in the RNASeq cohort, and a relatively stable clustering result was determined according to the CDF (the optimal number of clusters *k* = 3). Consistent clustering is a method based on resampling to verify the rationality of clustering. The method of resampling can disrupt the original dataset. Therefore, cluster analysis was performed on each resampled sample, and then multiple clusters were comprehensively evaluated. The result of the analysis reflected the consistency (Consensus), the main purpose of which was to assess the stability of clustering.

### Single-Sample Gene Set Enrichment Analysis

SsGSEA is an extension of gene set enrichment analysis (GSEA) (29). We applied GSVA R package (30) to calculate ssGSEA enrichment score representing the absolute enrichment degree of genes in a specific gene set for each sample. The gene expression values of a given sample ere sorted and normalized, and the empirical cumulative distribution function (ECDF) of the genes in the signature and the remaining genes was used to generate an enrichment score. To analyze the Th1/IFNγ expression differences in metabolic subtypes, we used the ssGSEA method to calculate the IFNγ score of each patient.

### Features of Immune Infiltration

To study the immune characteristics between different metabolic subtypes, we used the CIBERSORT method to evaluate the score distribution and differential expression of 22 immune cells in the TCGA dataset. CIBERSORT (31) is a tool for deconvolution of the expression matrix of immune cell subtypes based on the principle of linear support vector regression. Through the CIBERSORT function, the tissue transcriptome sequencing expression profile was statistically analyzed, and the de-convolution method was employed to denoise and remove unknown mixture content to estimate the relative proportion of 22 immune cell subpopulations. According to the expression profile data of each sequenced sample, we analyzed the relative expression of specific genes to predict the proportion of 22 kinds of immune cells.

### Prediction of Immunotherapy/Chemotherapy and Construct Subtype Characteristic Index

To compare the similarities between different metabolic subtypes and the GSE91061 dataset (melanoma dataset receiving anti-PD-1 and anti-CTLA-4 treatment) (32) among immunotherapy patients, we adopted a subclass mapping method SubMap analysis (33). SubMap analysis allows comparison of the similarity of the expression profiles of two independent datasets. We applied SubMap in the GenePattern software (http://www.broad.mit.edu/genepattern/) to analyze the similarity between different subtypes and GSE91061 dataset. The algorithm provides the calculation of a *p*-value to demonstrate the likelihood of molecular similarity between the different subclasses. At the same time, pRRophetic R package (34) was used to analyze the degree of response between different subtypes and four traditional chemotherapy drugs (Cisplatin, Paclitaxel, Embelin, Sorafenib).

In order to better quantify the immune characteristics of patients in different sample cohorts, linear discriminant analysis (LDA) (35) was employed to establish a subtype classification feature index. LDA is a linear method to fisher the induction of identification methods, the application of statistics, pattern recognition and machine learning methods, and to find a linear combination of the characteristics of two types of objects or events could help characterize or distinguish them. The resulting combination can serve as a linear classifier for dimensionality reduction processing for subsequent classification. Based on the LDA model, we calculated the subtype feature index of each patient in the TCGA dataset, and we can observe the feature index of different subtypes. Prognostic-related features in the TCGA dataset was used. First, we performed z-transformation on each feature and used Fisher's LDA optimization standard stipulated that the centroids of each group should be as dispersed as possible, and a linear combination A was found to maximize the between-class variance of A relative to the within-class variance. The characteristics of the model can distinguish samples of different subtypes for analysis.

### Weighted Correlation Network Analysis

The TCGA expression profile dataset (selecting MAD >50%) was selected. We used the R software package WGCNA (36) to cluster the samples, and screened the co-expression modules of metabolic genes (soft threshold = 4). Research showed that the co-expression network conformed to the scale-free network (log(k) and log(P(k)) were negatively correlated, and the correlation coefficient should be > 0.85. The expression matrix was further transformed into an adjacency matrix, and then the adjacency matrix was transformed into a topological overlap matrix (TOM). Based on TOM, we used the average-linkage hierarchical clustering method to cluster genes following the standard of hybrid dynamic shearing tree, and set the minimum number of genes for each gene network module to 100. When using dynamic shearing method, we determined the gene after the module, calculated the eigengenes of each module in turn, then performed cluster analysis on the modules, and merged the modules closer to each other into a new module (height=0.25, deepSplit = 2, minModuleSize = 100).

### Statistical Analysis

Statistical analysis was performed using R software (v3.6). For comparison of measurement data between groups, one-way analysis of variance or *t*-test was used for data conforming to normal distribution, Kruskal-Wallis H test or Mann-Whitney U test was used for non-normal data; Chi-square test or Fisher's exact probability method was used for supCount data. Difference analysis, test level α = 0.05; data with *p* < 0.05 was selected for analysis. ns, no significance. ^*^p < 0.05, ^**^p < 0.01, ^***^p < 0.001, ^****^p < 0.0001.

## Results

### Molecular Subtyping Based on Metabolic Gene Construction

**Figure 1:**
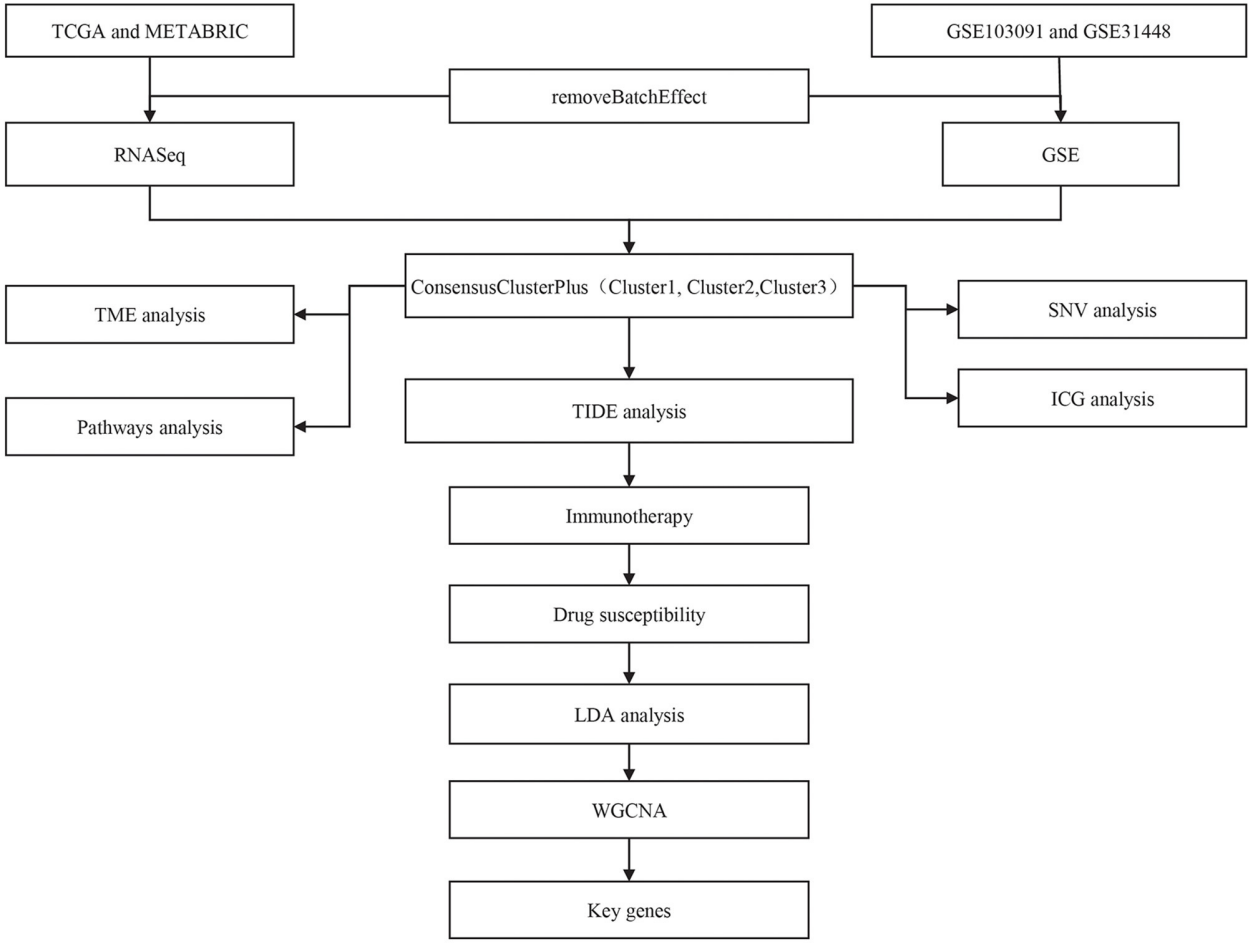
The workflow chart of this study.

The workflow of this study was shown in Figure 1. Principle component analysis (PCA) plots before and after data collection and elimination of batch effects (Supplementary Figure 1). After the two groups of data were preprocessed, 414 TCGA samples and 188 GSE samples were obtained.

**Figure 2:**
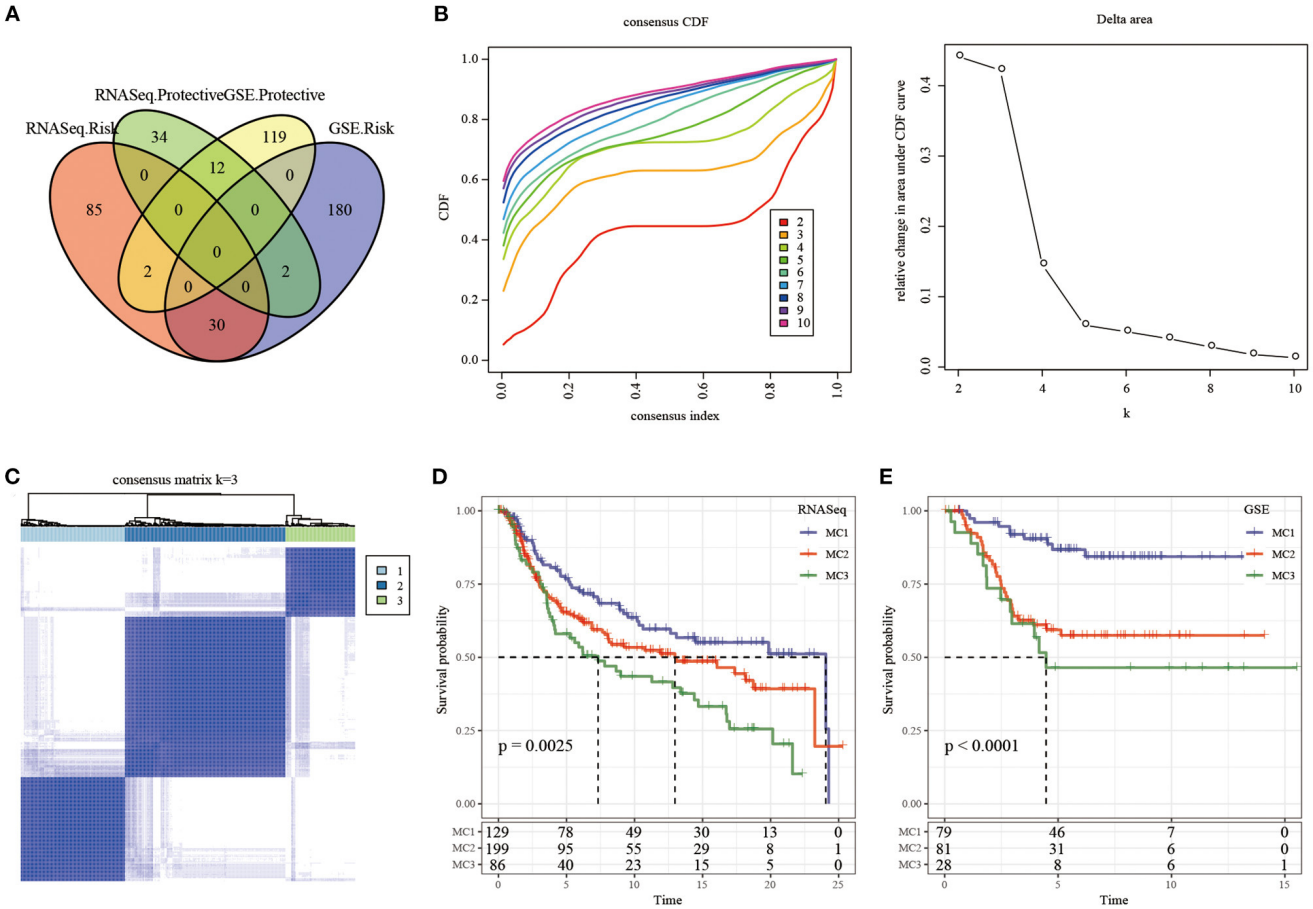
The Metabolism Cluster in TNBC. **(A)** Intersection Venn diagram of metabolic genes with significant prognosis in the two cohorts; **(B)** CDF curve of TCGA cohort samples and CDF Delta area curve, Delta area curve of consensus clustering, indicating the relative change in area under the cumulative distribution function (CDF) curve for each category number *k* compared with *k* – 1. The horizontal axis represents the category number k and the vertical axis represents the relative change in area under CDF curve; **(C)** consensus *k* =3 sample clustering heat map; **(D)** KM curve of the prognosis of the three subtypes in the RNASeq dataset; **(E)** KM curve of the prognosis of the three subtypes in the GSE dataset.

We first calculated the univariate analysis of metabolic genes from the two datasets. The univariate Cox regression analysis showed that a total of 165 genes and 345 genes were related to prognosis in the TCGA and GSE cohorts, respectively. The intersection number of genes between them was 42 (Figure 2A; Supplementary Table 1), which indicated that the consistency of metabolic genes was low among datasets of different platforms, and a single metabolic gene was quite different in different cohorts. Therefore, 42 metabolic genes were determined as prognostic-related genes for subsequent analysis (*p* < 0.05).

In the TCGA cohort, 414 TNBC samples were clustered by consensus clustering (ConsensusClusterPlus), and the optimal number of clusters was determined according to the CDF. By observing the CDF Delta area curve, it can be seen when the Cluster was selected as 3, there was a relatively stable clustering result (Figure 2B), and finally *k* = 3 was determined to obtain three metabolic subtypes (Metabolism Cluster, MC) (Figure 2C). Further analysis on the prognostic characteristics of these three metabolic subtypes showed that MC3 had a poor prognosis and MC1 had a better prognosis (Figure 2D, *p* = 0.0025). We used the same method in the GSE queue and obtained the same result (Figure 2E, *p* < 0.0001). The results showed that the three molecular subtypes based on metabolic genes were reproducible in different research cohorts.

### The Relationship Between Immunophenotyping and TMB and Common Gene Mutations

**Figure 3:**
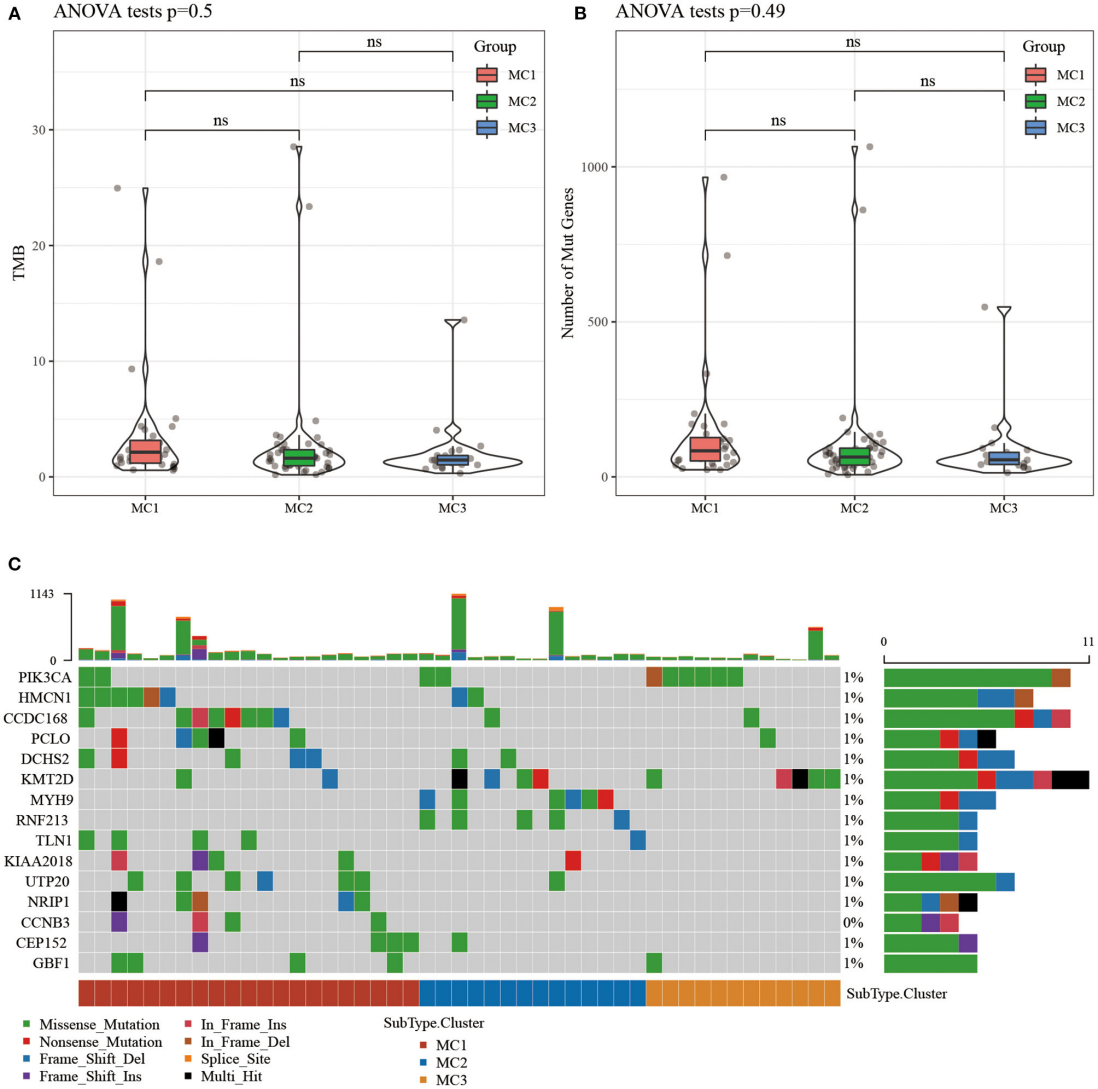
**(A)** The distribution difference of TMB in the three molecular subtypes of TCGA samples; **(B)** The distribution difference of the number of gene mutations in the three molecular subtypes of TCGA samples; use the rank sum test to determine the *p*-value, *p < 0.05; **p < 0.01; **(C)** The mutation characteristics of the significantly mutated gene in each subtype sample of TCGA dataset. ns, no significance.

We downloaded the TCGA mutation dataset (already processed by mutect2 software), calculated the tumor mutation burden (TMB) of TCGA patients, and analyzed the distribution of TMB in the three metabolic molecular subtypes (Figure 3A) and the difference in the number of mutant genes (Figure 3B). There was no statistical difference in the results. We further screened a total of 331 genes with mutation frequency >3% in sample percent, and used the chi-square test to identify genes with significant high frequency mutations in each subtype (threshold *p* < 0.05). Twenty-one genes remained and the top 15 mutated genes were visualized (Figure 3C).

### Expression of Chemokines and Immune Checkpoint Genes in Metabolic Typing

**Figure 4:**
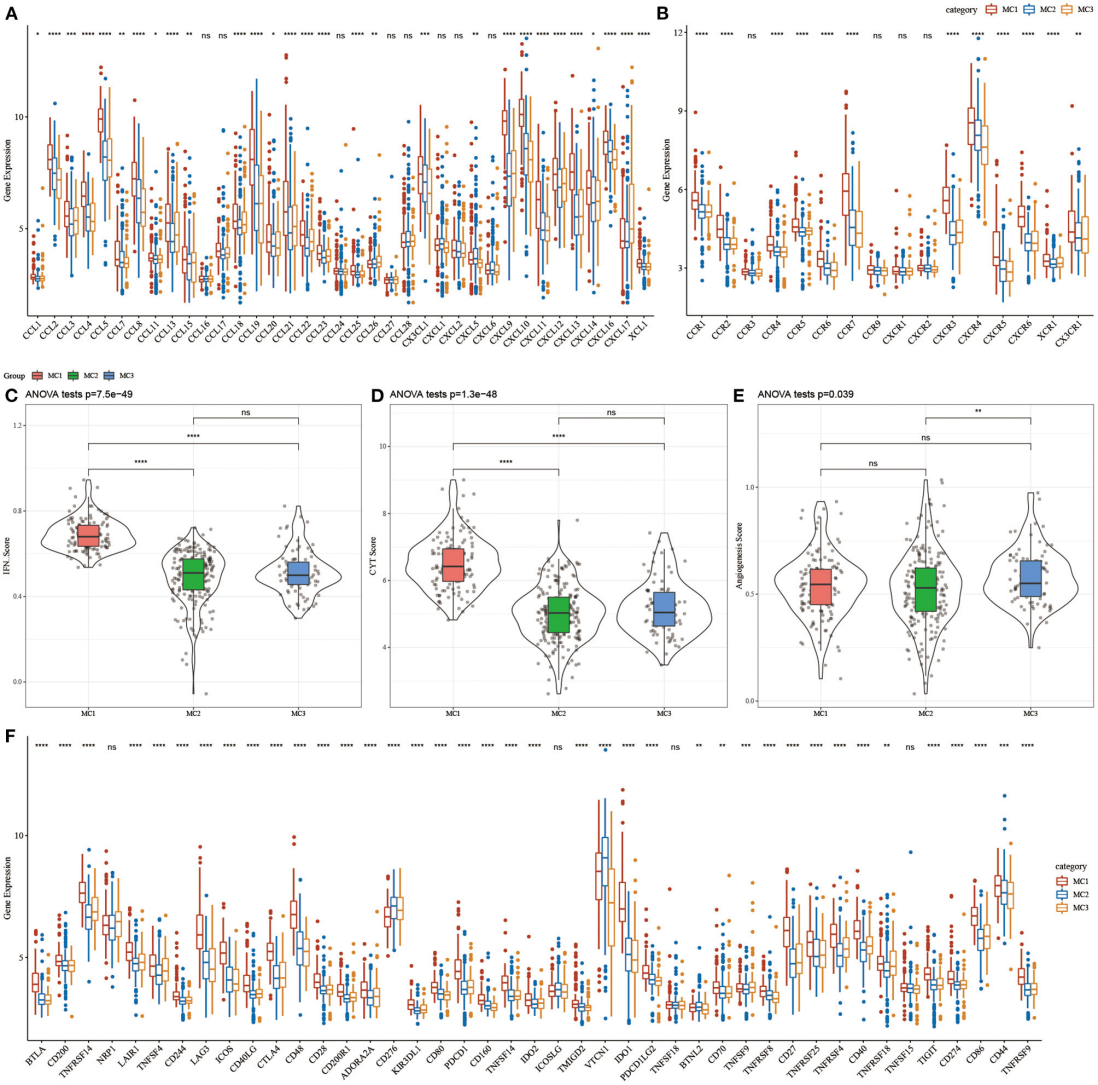
**(A)** Differences in the expression distribution of chemokines in the RNASeq cohort; **(B)** Differences in the expression and distribution of chemokine receptors in the RNASeq cohort; **(C)** Differences in the distribution of IFNγ scores of different subtypes in the RNASeq cohort; **(D)** Different subtypes Differences in immune T cell lysis activity; **(E)** differences in angiogenesis scores in different subtypes; **(F)** differences in the expression and distribution of immune checkpoint genes in the RNASeq cohort; the significance of which is statistically tested using analysis of variance, *p < 0.05; **p < 0.01, ***p < 0.001, ****p < 0.0001. ns, no significance.

To investigate whether there were differences in the expression of chemokines in the three metabolic subtypes, we calculated the differences in these genes in the TCGA cohort, and the results showed that 29 (78.34%) of the 37 chemokines existed in the subtypes. The significant difference (Figure 4A) indicates that the degree of immune cell infiltration of different metabolic subtypes may be different. In addition, we calculated and compared the expression of chemokine receptor genes in metabolic subtypes, and found that 12 (75%) out of the 16 chemokine receptor genes had significant differences in the expression of metabolic subtypes (Figure 4B).

To further study the difference in Th1/IFNγ expression between metabolic subtypes, we extracted Th1/IFNγ gene signatures from previous studies (37), and calculated the IFNγ score of each patient using the ssGSEA method. The results showed that IFNγ in each subtype, there were significant differences in the scores. MC1 subtypes had higher IFNγ scores, while MC2 and MC3 subtypes had the lowest IFNγ scores (Figure 4C).

To analyze the difference in immune T cell lysis activity between the metabolic subtypes, according to the study of Rooney Michael S (38), the average value of GZMA and PRF1 expression levels was used to evaluate the immune T cell lysis activity in each patient's tumor. There were significant differences in these subtypes (*p* < 0.001). Interestingly, MC1 had the highest immune T cell lytic activity, while MC2 and MC3 had the lowest immune T cell cytotoxic (CYT) activity (Figure 4D).

The difference in angiogenesis scores between the metabolic subtypes was studied. The angiogenesis-related gene set was obtained from previous studies, and the angiogenesis scores of each patient were evaluated. The results showed significant differences in different subtypes. The angiogenesis score of MC3 was significantly higher than that of MC2 (Figure 4E).

In order to study the differences in immune checkpoint-related genes among various metabolic subtypes, we obtained 43 immune checkpoint-related genes from previous studies (37). The data demonstrated that 37 (90.70%) genes had significant differences. It was observed that the expression of most immune checkpoint-related genes in MC1 was significantly higher than that in MC3.

### Immune Characteristics and Pathway Characteristics in Different Metabolic Subtypes

**Figure 5:**
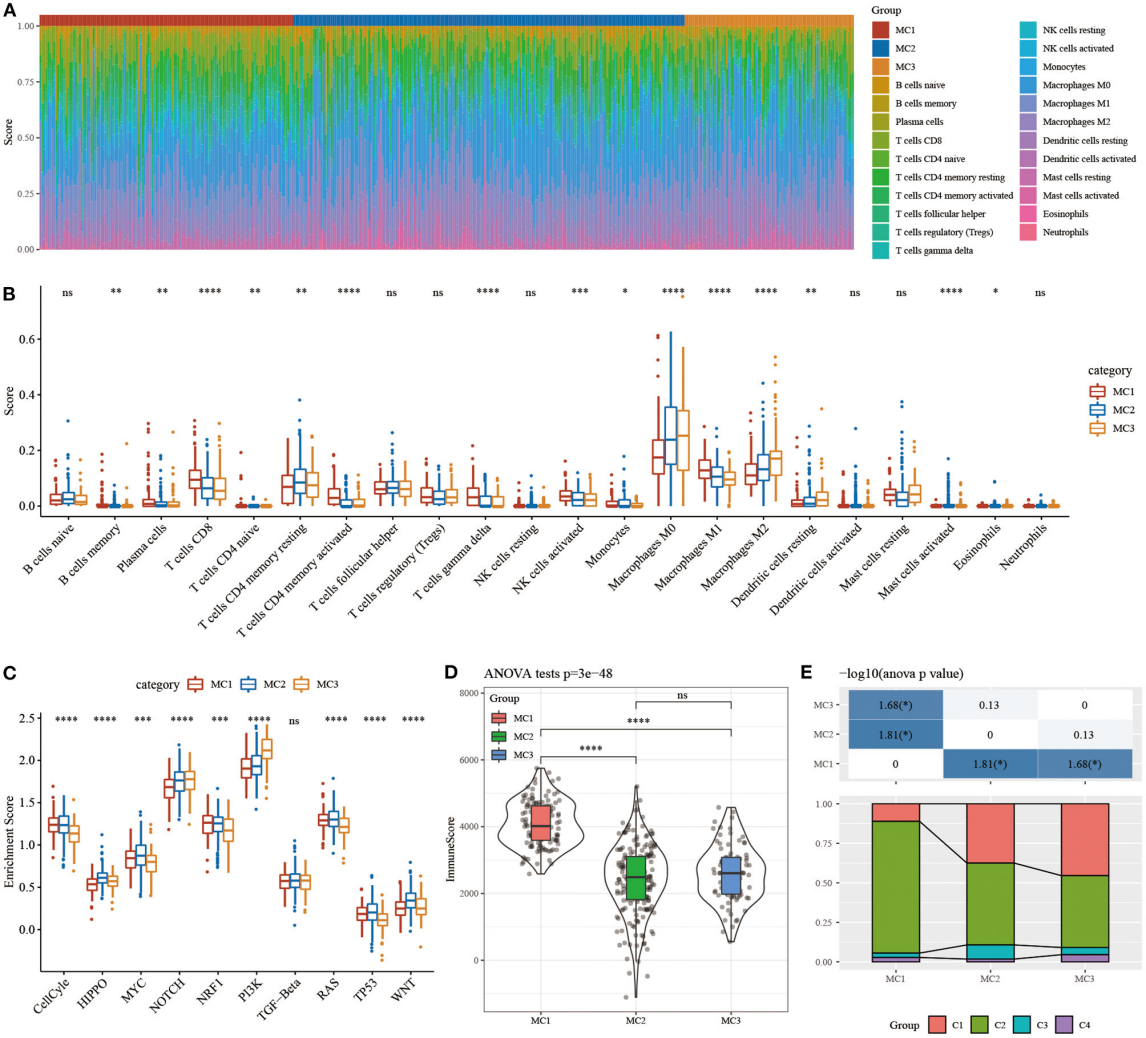
**(A)** The ratio of 22 immune cell components in samples of different subtypes. **(B)** Differences in 22 immune cell components of samples in different subtypes; **(C)** Differences in scores of 10 pathways related to tumor abnormalities in different subtypes; **(D)** Differences in immune infiltration scores in different subtypes. **(E)** Comparison of the three metabolic molecular subtypes with the previous six pan-cancer metabolic molecular subtypes. ns, no significance. *P < 0.05, **P < 0.01, ***P < 0.001, ****P < 0.0001.

In order to study the immune characteristics between different metabolic subtypes, we used the CIBERSORT method in the RNASeq dataset to evaluate the scores of 22 immune cells in each sample, and observe the distribution of immune cell scores in the three subtypes (Figure 5A) and immune cell scores. The difference result (Figure 5B) found that there are significant differences in immune characteristics among the subtypes: CD8 T cells, resting memory CD4 T cells, macrophages M0, macrophages M1, macrophages M2, etc. are all significantly higher in the subtypes, indicating that they may play an important role in triple-negative breast cancer. In order to study the characteristics of the pathways in each subtype, 10 oncogenic pathways in the previous study (39) were obtained, and the results showed that 9 of the 10 pathways had significant differences between the subtypes (Figure 5C). The analysis of immune infiltration showed that MC1 had the highest immune microenvironment infiltration, whereas MC2 and MC3 had the lowest immune infiltration scores (Figure 5D).

The relationship between metabolic subtypes and the six previous pan-cancer immunotypes was analyzed, and we extracted the molecular subtype data of these samples from previous studies for comparison. We observed significant differences in the previous pan-cancer immunotypes (Figure 5E; Supplementary Figure 2). There was no difference between the survival curves of TNBC samples in pan-cancer immunophenotyping. The analysis suggested that these three subtypes can be used as a supplement beyond the six subtypes in the previous study.

### Analysis of the Difference of TIDE Among Metabolic Subtypes

**Figure 6:**
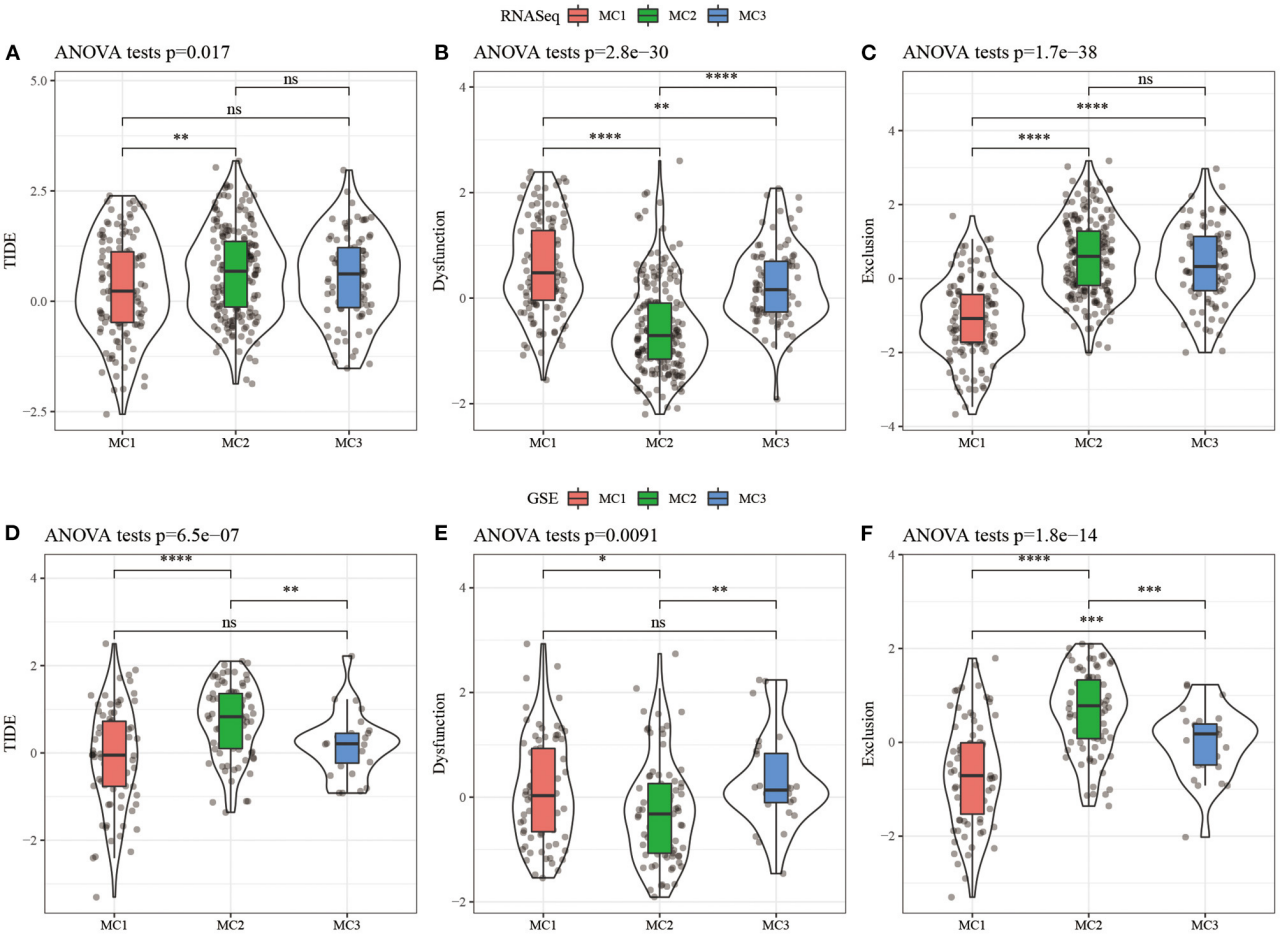
**(A)** TIDE score difference of different metabolic subtypes of RNASeq; **(B)** T cell dysfunction score difference of different metabolic subtypes of RNASeq; **(C)** T cell rejection score difference of different metabolic subtypes of RNASeq; **(D)** Different metabolic subtypes of GSE TIDE score difference; **(E)** HCCDB18 different metabolic subtypes of T cell dysfunction score difference; **(F)** GSE different metabolic subtypes of T cell rejection score difference. ns, no significance. *P < 0.05, **P < 0.01, ***P < 0.001, ****P < 0.0001.

In order to study the differences of different metabolic molecular subtypes in immunotherapy, we use TIDE software (http://tide.dfci.harvard.edu/) (40) to evaluate the potential clinical effects of immunotherapy on metabolic molecular subtypes. Higher TIDE prediction score was correlated with a higher possibility of immune escape, indicating that the patient was less likely to benefit from immunotherapy. The results showed that in the RNASeq dataset, the TIDE score of MC2 was significantly higher than that of MC1, suggesting that MC1 can benefit from immunotherapy more than MC2 (Figure 6A). At the same time, we also compared the differences in the predictive T cell dysfunction scores and T cell rejection scores of metabolic molecular subtypes. We found that compared to MC1 and MC3, MC2 had lower predicted T cell dysfunction scores (Figures 6B,C). In comparing the results of predicted T cell rejection scores, it was found that MC2 had a higher T cell rejection score, while MC1 had a lower T cell rejection score, moreover, we also observed similar results on the GSE dataset (Figures 6D–F).

### Analysis of the Difference Between Metabolic Subtypes and Immunotherapy/Chemotherapy

**Figure 7:**
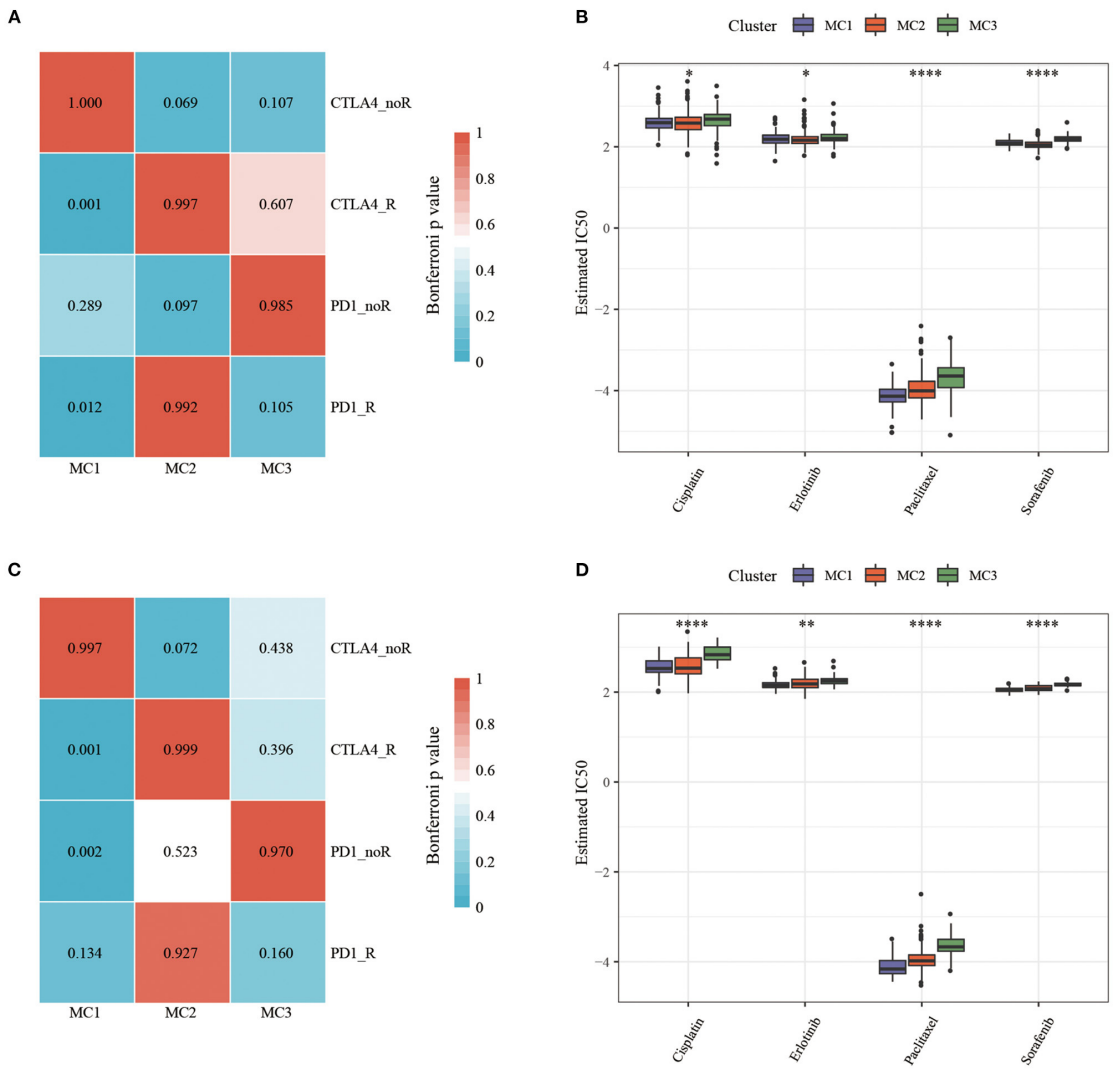
**(A)** RNASeq, Submap analysis manifested that MC1 could be more sensitive to CTLA4 (Bonferroni-corrected *P* < 0.05); **(B)** RNASeq, The box plots of the estimated IC50; **(C)** GSE, Submap analysis manifested that MC1 could be more sensitive to CTLA4 (Bonferroni-corrected *P* < 0.05); **(D)** GSE, The box plots of the estimated IC50. *P < 0.05, **P < 0.01, ****P < 0.0001.

In order to study the differences in immunotherapy and chemotherapy of different immune molecular subtypes, we used the method of subclass mapping to compare the three metabolic subtypes with the GSE91061 dataset (melanoma receiving anti-PD-1 and anti-CTLA-4 treatment). The similarity between immunotherapy patients in the dataset was analyzed, and we found that lower *p*-value was correlated with higher similarity. The results showed that the MC1 subtype was more sensitive to CTLA4 inhibitors than the other two subtypes (Figures 7A,C). At the same time, we also analyzed the response of different subtypes to the traditional chemotherapy drugs Cisplatin, Paclitaxel, Embelin, and Sorafenib, and found that the MC1 subtype was more sensitive to these four drugs than other subtypes (Figures 7B,D).

### LDA and Metabolic Subtype Characteristic Index Construction

**Figure 8:**
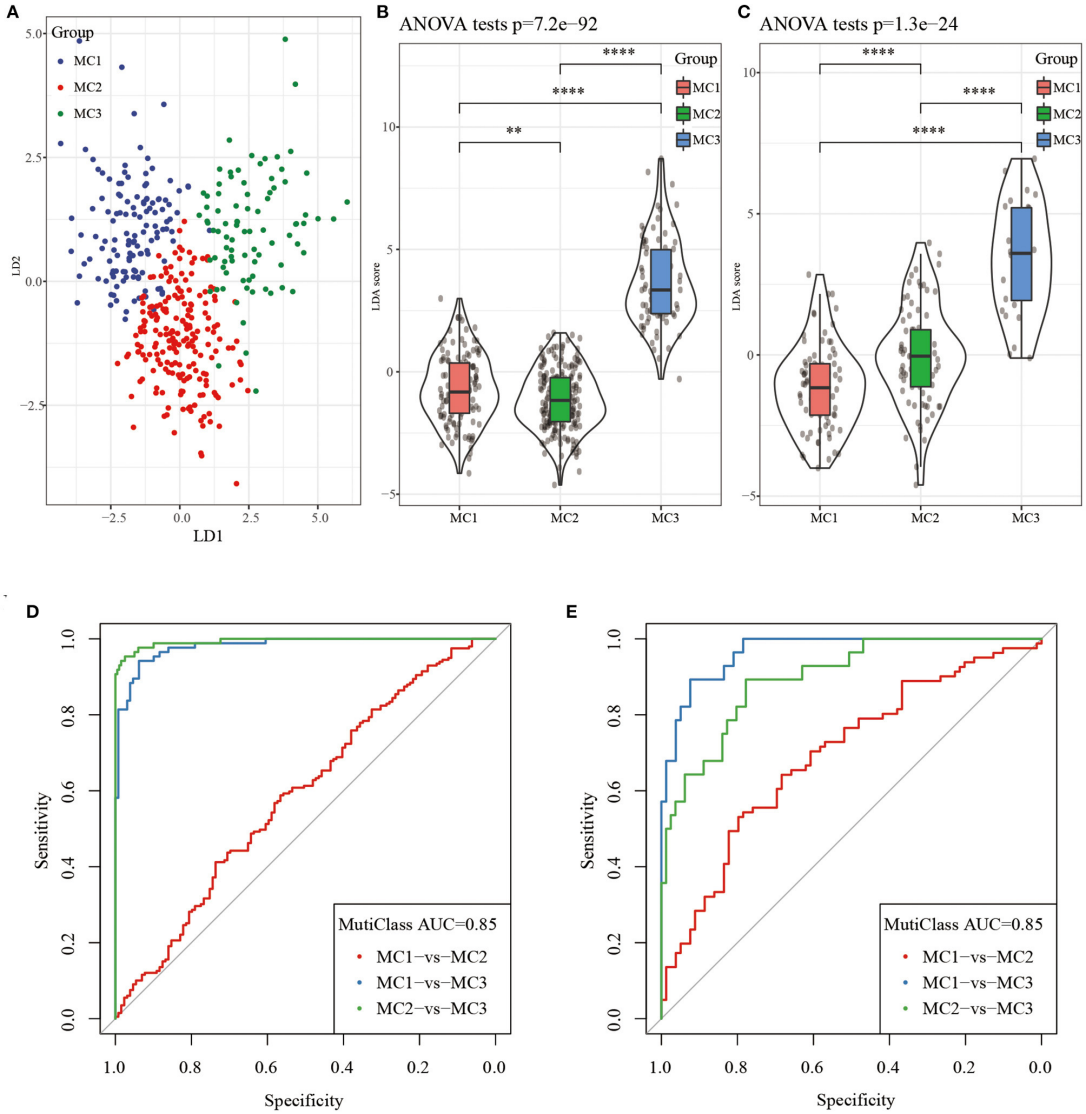
**(A)** The relationship between the first two features in the RNASeq metabolic feature index and the metabolic subtypes, **(B)** The difference in the metabolic feature index of different subtypes in the RNASeq dataset; **(C)** The difference in the metabolic feature index of different subtypes in the GSE dataset; **(D)** ROC curve of metabolic feature index in RNASeq dataset; **(E)** ROC curve of metabolic feature index in GSE dataset. **P < 0.01, ****P < 0.0001.

Considering that different subtypes have different molecular characteristics, to better quantify the immune characteristics of patients in different cohorts, we used LDA to establish a subtype classification feature index. LDA can be used as a supervised dimensionality reduction technology, which is often suitable for multiple types of problems. Specifically, we used 42 prognostic-related genes in the RNASeq dataset, first performed z-transformation on each feature, and then used Fisher's LDA optimization standard to stipulate that the centroid of each group should be exhausted. The first two features of the model can clearly distinguish samples of different subtypes (Figure 8A). Based on the LDA model, the subtype feature index of each patient was calculated in the RNASeq dataset. Significant differences in the feature index of different subtypes can be observed (Figure 8B). ROC analysis showed the classification performance of the feature index in different subtypes (Figure 8D), the multi-category comprehensive forecast AUC was 0.85. Applying the metabolic subtype feature index to the GSE dataset, we found that the results were similar to the RNASeq dataset. There were significant differences in the feature index of different subtypes (Figure 8C). ROC analysis showed that the comprehensive AUC was 0.85 (Figure 8E).

### Identification of Metabolic Characteristic Index Co-expressed Gene Modules

**Figure 9:**
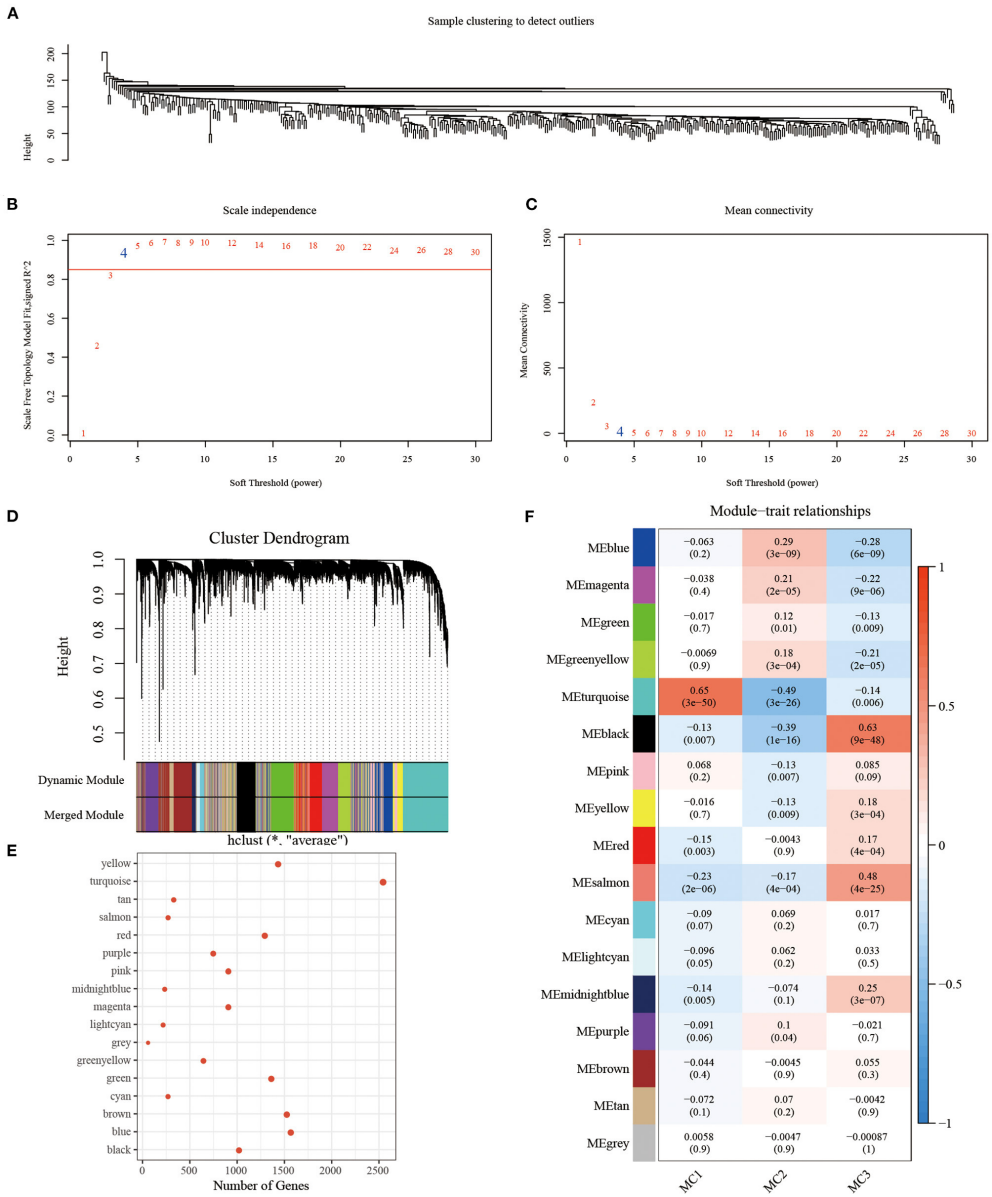
**(A)** Clustering tree of each sample; **(B)** Analysis of the scale-free fit index for various soft-thresholding powers (β). **(C)** Analysis of the mean connectivity for various soft-thresholding powers. **(D)** Dendrogram of all differentially expressed genes/lncRNAs clustered based on a dissimilarity measure (1-TOM); **(E)** statistics of the number of genes in each module; **(F)** correlation between each module and subtype.

In order to identify the co-expression modules of these genes, we selected the RNASeq expression profile dataset, and used the R software package WGCNA to cluster the samples to screen the co-expression modules (soft threshold = 4, Figure 9A). Research showed that the co-expression network conforms to the scale-free network [log(k) was negatively correlated with log(P(k)], and the correlation coefficient was >0.85). β = 4 was set to ensure that the network was scale-free (Figures 9B,C). Next, the expression matrix was converted into an adjacency matrix, and then the adjacency matrix was converted into a topological matrix. Based on TOM, we used the average-linkage hierarchical clustering method to cluster genes, according to the standard of hybrid dynamic shearing tree, and set each the minimum number of genes in a gene network module to be 100. After determining the gene modules using the dynamic shearing method, we calculated the eigengenes of each module in turn, then performed cluster analysis on the modules, and merged the modules close to each other into a new module (height=0.25, deepSplit = 2, minModuleSize = 100). Here, a total of 17 modules were obtained, of which the gray module was a gene module that cannot be allocated (Figures 9D,E). We analyzed the correlation between each module and MC1, MC2, and MC3 (Figure 9F). Among them, the correlation between turquoise and black modules and MC1 and MC3 was >0.6.

### Identification of Metabolic Characteristic Index Co-expressed Gene Modules and Prognostic Analysis

**Figure 10:**
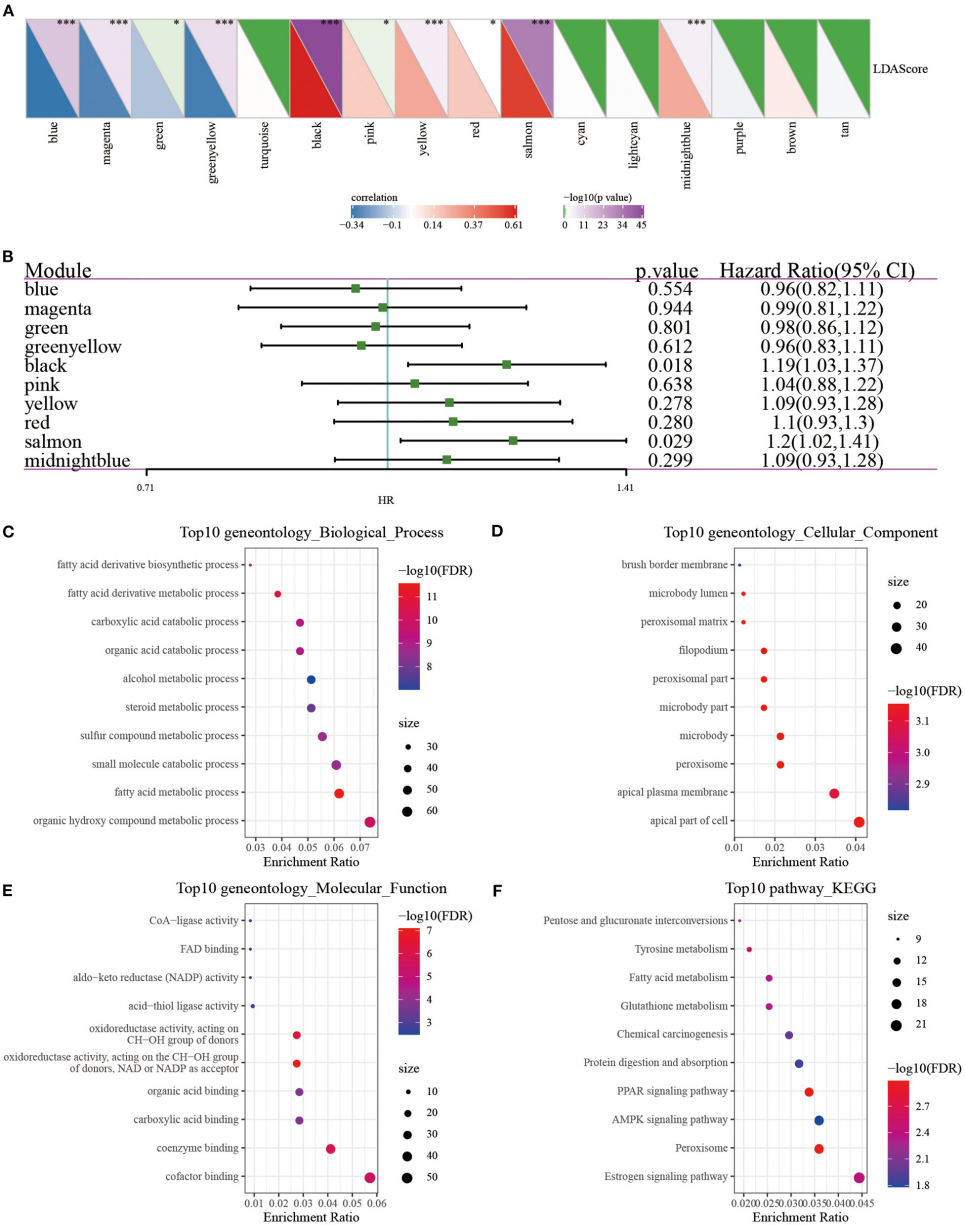
**(A)** Correlation analysis of LDA score and metabolic characteristic index; **(B)** Prognostic correlation of modules related to metabolic characteristic index; **(C–F)** GO and KEGG analysis of black module genes. *P < 0.05, ***P < 0.001.

We, respectively, calculated the correlation between the feature vector of these 17 modules and the metabolic feature index, and it can be seen that there were 17 modules significantly related to the metabolic feature index (Figure 10A). Furthermore, we selected modules significantly related to the metabolic characteristic index for prognostic analysis. According to the relationship between the module and the metabolic molecular subtype and the relationship between the module and the prognosis, we further screened the black module (Figure 10B), according to the correlation coefficient of the module feature vector > genes with 0.7. Significant prognosis were used as the hub-genes (threshold *p* < 0.05), and finally seven key genes (***CLCA2, HGD, REEP6, SPDEF, ABCC11, SPINK8, CRAT***) were identified in the black module. These seven genes were found to be differentially expressed in three subtypes, and MC3 had the highest expression of all seven genes than MC1 and MC2 (*P* < 0.0001, Supplementary Figure 3), which further demonstrated that the seven key genes may be closely involved in the TNBC development. In addition, protein-protein interaction (PPI) analysis using STRING showed that five of seven genes (*HGD, REEP6, SPDEF, ABCC11*, and *CRAT*) interacted with at least two other genes (Supplementary Figure 4). CRAT had the greatest number of interacted genes closely involved in metabolism-related pathways such as fatty acid metabolism and fatty acid degradation (Supplementary Table 2).

In order to analyze whether there was a difference in prognosis between high and low gene expression groups, we divided patients into high and low expression groups according to gene expression. The results demonstrated that the survival curves of genes ***CLCA2, REEP6*,** ***SPDEF*, and** ***CRAT*** were significantly different (Supplementary Figure 5). However, in other breast cancer subtypes of basal, her2, luminal A, luminal B and normal, we observed that the overall survival of groups of with high and low expression of the seven genes was less significantly different (Supplementary Figure 6), suggesting that these seven genes could be more specifically applied to the TNBC patients. Next, we used the clusterProfiler package (41) to enrich the genes of the black module (Figures 10C–F). It can be observed that the black module was closely related to metabolic processes such as Tyrosine metabolism, Fatty acid metabolism, PPAR signaling pathway, and Glutathione metabolism.

## Discussion

Breast cancer is a common malignant tumor in women. In 2020, there were ~2.3 million newly diagnosed breast cancer cases worldwide, accounting for 11.7% of all new cancer cases and mortality rate of 6.9%. It is one of the main causes of female cancer deaths (42). Breast cancer is increasingly recognized as a highly heterogeneous disease, showing great differences in pathological characteristics, biological behavior and gene expression profiles (43). According to St. Gallen International Conference on Breast Cancer in 2015 (44), based on the estrogen receptor (ER) progesterone receptor (PR), human epidermal growth factor receptor-2 (human epidermalgrowth factor), the expression of receptor-2, HER2) and Ki-67 proliferation index in tumor tissues is different, and breast cancer is divided into 4 molecular subtypes. TNBC accounts for about 15–20% of all breast cancer subtypes (45). Due to a lack of expression of ER, PR and HER2, breast cancer lacks precise molecular therapeutic targets, and surgery supplemented with radiotherapy and chemotherapy is the main treatment method (46). However, its heterogeneity and invasiveness are strong, recurrence is early and the rate of visceral metastasis is high. Once recurrence and metastasis occur, the median survival period is generally shorter than 1 year. The prognosis of other subtypes is worse (47).

As TNBC patients show different treatment responses and prognosis, individualized treatment and prognostic analysis of TNBC patients could be difficult, especially when the diagnosis and treatment of TNBC patients depends on routine clinical and pathological characteristics (including histological grade, primary tumor size, lymph node metastasis and estrogen receptor/progesterone receptor/HER2 expression). To improve the survival and prognosis of TNBC patients, in recent years, the exploration of the heterogeneity and molecular characteristics of TNBC has been gradually deepened. Deciphering the characteristics of different subtypes of TNBC through molecular typing can provide evidence for the early diagnosis and prognosis of TNBC, a necessary prerequisite for individualized targeted therapy.

In current studies on the classification of triple-negative breast cancer, Lehmann et al. (48) identified six TNBC subtypes based on gene expression profiles, including two basal-like subtypes (BL-1 and BL-2), immunomodulatory subtype (IM), mesenchymal subtype (M), mesenchymal stem cell-like subtype (MSL), and luminal/androgen receptor subtype (LAR). Subsequent studies conducted by Masuda et al. (49) further confirmed the clinical significance of Lehmann classification and added an unstable subtype (UNS). Bonsang-Kitzis et al. (50) identified six TNBC subtypes including two immune clusters based on a biological network-driven method. Their matrix immune module gene signatures showed a strong prognostic value. Burstein et al. (51) identified four stable TNBC subtypes based on mRNA expression and DNA genome analysis, Luminal/Androgen receptor type (LAR), mesenchymal type (MES), basal-like immunosuppressive type (BLIS) and Basal-like immune activation (BLIA), and determined potential therapeutic targets for these specific subtypes. These typing studies on TNBC lay the foundation for the exploration of targeted therapeutic targets. However, few studies have specifically explored the TNBC classification based on metabolic characteristics. Therefore, exploring the TNBC classification based on metabolic characteristics may help TNBC patients to achieve the optimal stratification in clinical treatment to achieve the role of precise treatment.

In the present study, we typed TNBC at the metabolic molecular level, and found that based on 2,752 metabolic genes to type TNBC, these samples can be divided into three metabolic subtypes (MC1, MC2, MC3), and that the subtypes showed significant differences in prognosis, specifically, MC1 had a better prognosis, while MC3 had a poor prognosis (Figure 2). Different metabolic subtypes had different immune characteristics, which may be useful for immunotherapy. There were different response patterns (Figures 4, 5) in different research queues, metabolic subtypes had a high degree of reproducibility. Based on metabolic subtypes, an immune characteristic index has been established, which can better quantify the immunity of patients' characteristics, reflecting different degrees of immune infiltration of patients. Metabolic characteristic index was related to immune checkpoint. Finally, based on the co-expression network analysis, we screened seven potential gene markers related to the metabolic characteristic index. Among them, the differential expression of 4 genes including ***CLCA2, REEP6, SPDEF, and CRAT*** had significant significance for the prognosis of TNBC.

Recent studies have shown that a variety of tumor cells, including triple-negative breast cancer, can affect the tumor immune microenvironment (TIME) by stimulating cancer-promoting inflammation, sending out immunosuppressive signals and evading immune surveillance (52). TIME promotes tumor progression and metastasis by promoting tumor angiogenesis, influencing tumor biological characteristics, screening host cells and regulating tumor stem cell activity. Its main cell components include T lymphocytes, B lymphocytes, macrophages, NK cells and DC cells. Among them, the T and B cells, which circulate through the peripheral blood and exist in tumor tissues and local microenvironment, are collectively called tumor infiltrating lymphocytes (TIL). This type of cell group participates in and mediates the occurrence and development of tumors, indicating that patients may have immune response of malignant tumors. The presence of TIL have been confirmed to be related to favorable prognosis of TNBC and HER2-positive breast cancer and active response to chemotherapy (53). In our research, we found that different metabolic subtypes had significant differences in the expression of chemokines and their receptor genes. These differential expressions indicated that different metabolic subtypes had different degrees of immune cell infiltration, which may lead to tumor progression and differences in the effectiveness of immunotherapy. At the same time, tumor-related cytokines and chemokines can recruit and polarize immune subpopulations and differentiate into pro-tumor phenotypes, thereby promoting tumorigenesis.

We analyzed the infiltration of 22 types of immune cells in triple-negative breast cancer samples, and the results showed that T cells and various types of macrophages (M0, M1, M2) were significantly high-expressed in each subtype. Regarding the progression of macrophages in TNBC, Sami E [56] and others believe that macrophages promote the aggressiveness of breast cancer by promoting its M2 polarization, leading to a poor prognosis. At the same time, CD8 + T cells in the tumor microenvironment can produce IFN-γ, which in turn stimulates the up-regulation of PD-1/PD-L1 and IDO1 gene expression (54). Studies have shown that the up-regulation of PD-L1 expression in tumor cells, especially when combined with PD-1 expressed by tumor infiltrating activated T cells, can induce exhaustion and inhibit the anti-tumor immune activity of these effector cells, thereby allowing tumor cells to escape from immunity (55). The up-regulation of IDO1 expression is positively correlated with poor prognosis and tumor progression and metastasis (56, 57).

In our research, we calculated the IFNγ score, immune T cell lytic activity, angiogenesis score, and immune checkpoint-related gene expression in the three metabolic subtypes. Based on the above results, we found that the MC1 subtype with the optimal prognosis had higher IFNγ score and T cell lytic activity, lower angiogenesis score and TIDE score, indicating that this subtype had stronger immunogenicity and better tumor microenvironment, and was more likely to benefit from immunotherapy. In the differential analysis of immune checkpoint expression in different subtypes, we observed that the expression of most immune checkpoint-related genes (LAG3, CTLA4, PDCD1, CD276, HAVCR2) in MC1 was significantly increased, at the same time, compared with the other two subtypes, MC1 subtype was more sensitive to immune checkpoint inhibitors (CTLA-4) and traditional chemotherapy drugs (Cisplatin, Paclitaxel, Embelin, Sorafenib). This further confirmed our findings. The well-known IM passion130 study has confirmed the effectiveness of immune checkpoint inhibitors in the treatment of breast cancer (58). In particular, immune checkpoint inhibitors combined with standard chemotherapy regimens can significantly prolong the treatment of TNBC patients compared with using standard chemotherapy regimens alone.

Increasing evidence showed that epigenetic changes play an important role in the pathogenesis of cancer. There are research results reporting the epigenetic changes related to the clinical prognosis of TNBC, which also increase the complexity of TNBC molecular typing. Although the TNBC molecular prognostic evaluation model has broad clinical application prospects and related research results have been verified to a certain extent, there is currently no unified and widely recognized molecular prognostic evaluation model in clinical practice. At present, there are still controversies about the scope of application of the molecular prognostic assessment model. All established prognostic models require large sample verification and clinical application research, which is a huge task at the current stage. And when a prognostic model is not well applicable to new populations, the new data and re-calibration should be used to adjust the model and to improve its stability and adaptability. Only through verification-adjustment-re-verification, the molecular prognostic model obtained may be reliable and accurate (59).

The research on molecular typing and individualized treatment of TNBC started late, but according to the existing clinical evidence, molecular typing can be correlated with individualized treatment and can become one of the effective methods of TNBC treatment. In short, the clinical diagnosis and treatment of TNBC in the near future will be based on molecular prognostic model, molecular classification and prognostic evaluation, and then individualized molecular therapy will be carried out, which will significantly improve the therapeutic effect of TNBC as well as the survival and prognosis of patients.

This study identified seven key genes, and CLCA2, REEP6, SPDEF, and CRAT were significantly associating with prognosis. Calcium-activated chloride channel 2 (CLCA2) expression was reported to be significantly downregulated in cervical squamous cell carcinoma, and was found to be negatively correlated with the enrichment of immune cells, especially with B cells, macrophage cells, and dendritic cell (60). Purrington demonstrated that CLCA2 expression was associated with TNBC overall survival (HR = 1.56, 95% CI = 1.31–1.86) in African American women (61). SAM pointed domain ETS factor (SPDEF) was considered to have both oncogenic and tumor-suppressive effects in breast cancer (62). CRAT gene was less reported in cancer research and immune microenvironment, but it was identified as prognostic genes in bladder cancer (63). These four key metabolic genes related to TNBC prognosis may serve as novel research targets for understanding their metabolic mechanisms in TNBC development.

In conclusion, this study established a metabolic classification as an independent prognostic factor for TNBC, and analyzed the differences in the characteristics of tumor immune microenvironment between subtypes, so as to measure the prognostic risk of TNBC patients, guide clinical diagnosis, staging, and individualized treatment, and support prognostic prediction.

## Data Availability Statement

The datasets presented in this study can be found in online repositories. The names of the repository/repositories and accession number(s) can be found in the article/Supplementary Material.

## Ethics Statement

The Ethics Committee approved this study of the Jiamusi Medical University of 1st Affiliated Hospital.

## Author Contributions

YZ and YC: conception and design and acquisition of data. YZ, YC, and ZF: analysis and interpretation of data. YC, ZF, and HZ: writing, review, and revision of the manuscript. HW: study supervision. All authors read and approved the final manuscript.

## Conflict of Interest

The authors declare that the research was conducted in the absence of any commercial or financial relationships that could be construed as a potential conflict of interest.

## Publisher's Note

All claims expressed in this article are solely those of the authors and do not necessarily represent those of their affiliated organizations, or those of the publisher, the editors and the reviewers. Any product that may be evaluated in this article, or claim that may be made by its manufacturer, is not guaranteed or endorsed by the publisher.

## Supplementary Material

Supplementary Figure 1 Batch effect removal. **(A,B)** PCA chart before and after batch effect removal from RNASeq data; **(C,D)** PCA charts before and after batch effect removal from GSE data.

Supplementary Figure 2 The survival status of immune subtypes.

Supplementary Figure 3 The expression of seven key genes in three subtypes in the RNASeq dataset. Kruskal-Wallis test was performed. ^****^*P* < 0.0001.

Supplementary Figure 4 The PPI results of five genes (CLCA2 and SPINK8 were not shown as no interaction networks were found).

Supplementary Figure 5 Four genes with survival differences.

Supplementary Figure 6 Kaplan-Meier survival curves of five subtypes of breast cancer including basal **(A)**, her2 **(B)**, luminal A **(C)**, luminal B **(D)**, and normal **(E)** grouping by high and low expression groups of the seven key genes.

Supplementary Table 1 The 42 co-significant genes between RNASeq and GSE cohorts were collected.

Supplementary Table 2 The enriched pathways of interacted genes.
