# Supplementary figures and images for: Triple-Negative Breast Cancer Analysis Based on Metabolic Gene Classification and Immunotherapy
Source: Front Public Health. 2022 Jul 6;10:902378. doi: 10.3389/fpubh.2022.902378 (PMC9296841; doi:10.3389/fpubh.2022.902378)

A

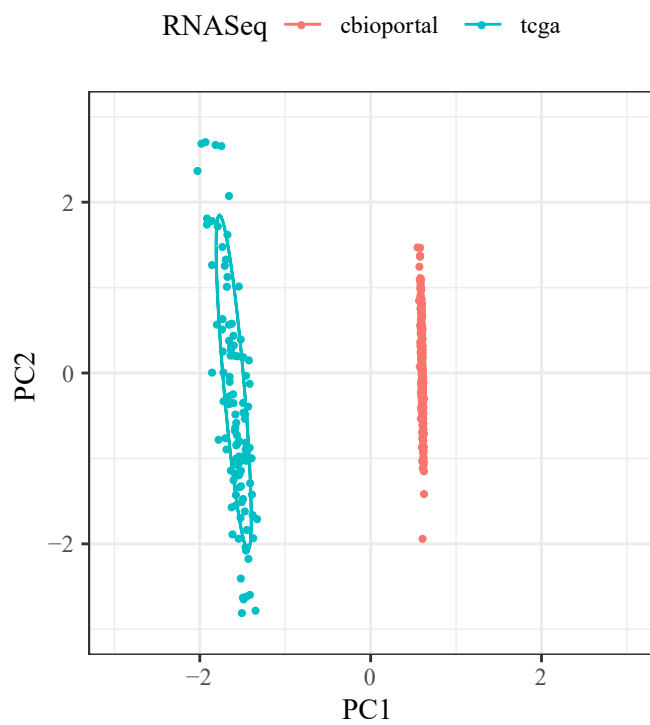

B

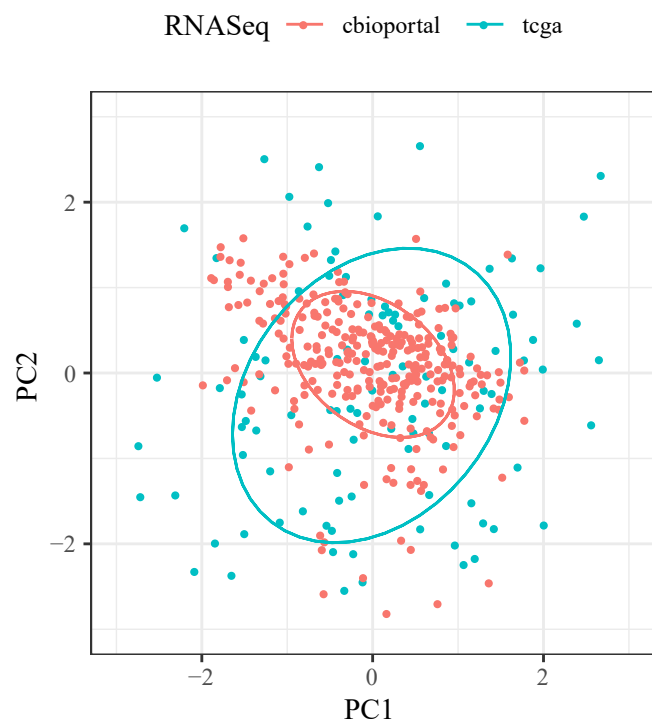

C

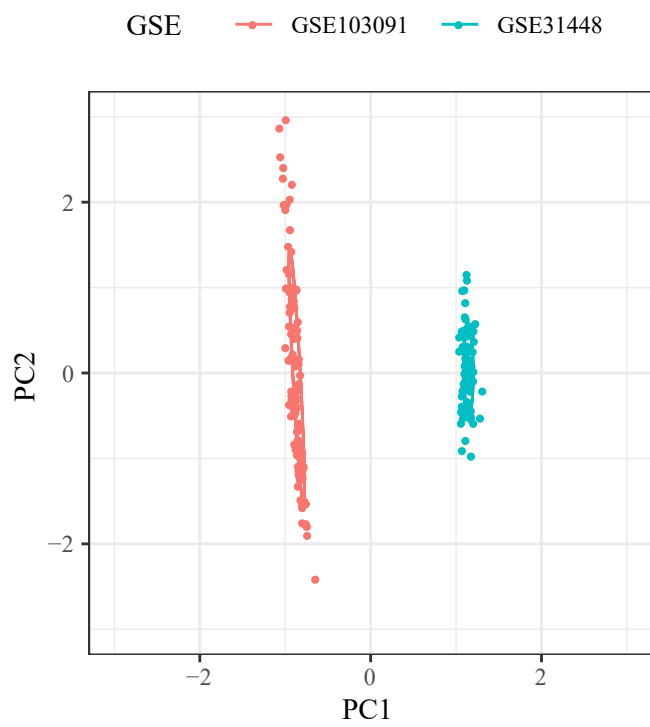

D

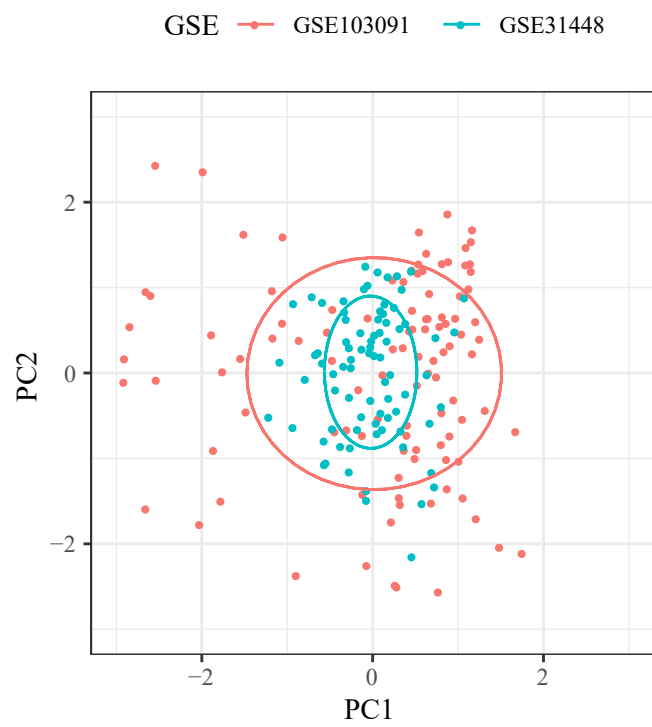

Supplement: Supplementary Figure 1 — Batch effect removal. (A,B) PCA chart before and after batch effect removal from RNASeq data; (C,D) PCA charts before and after batch effect removal from GSE data. [file Image_1.pdf]

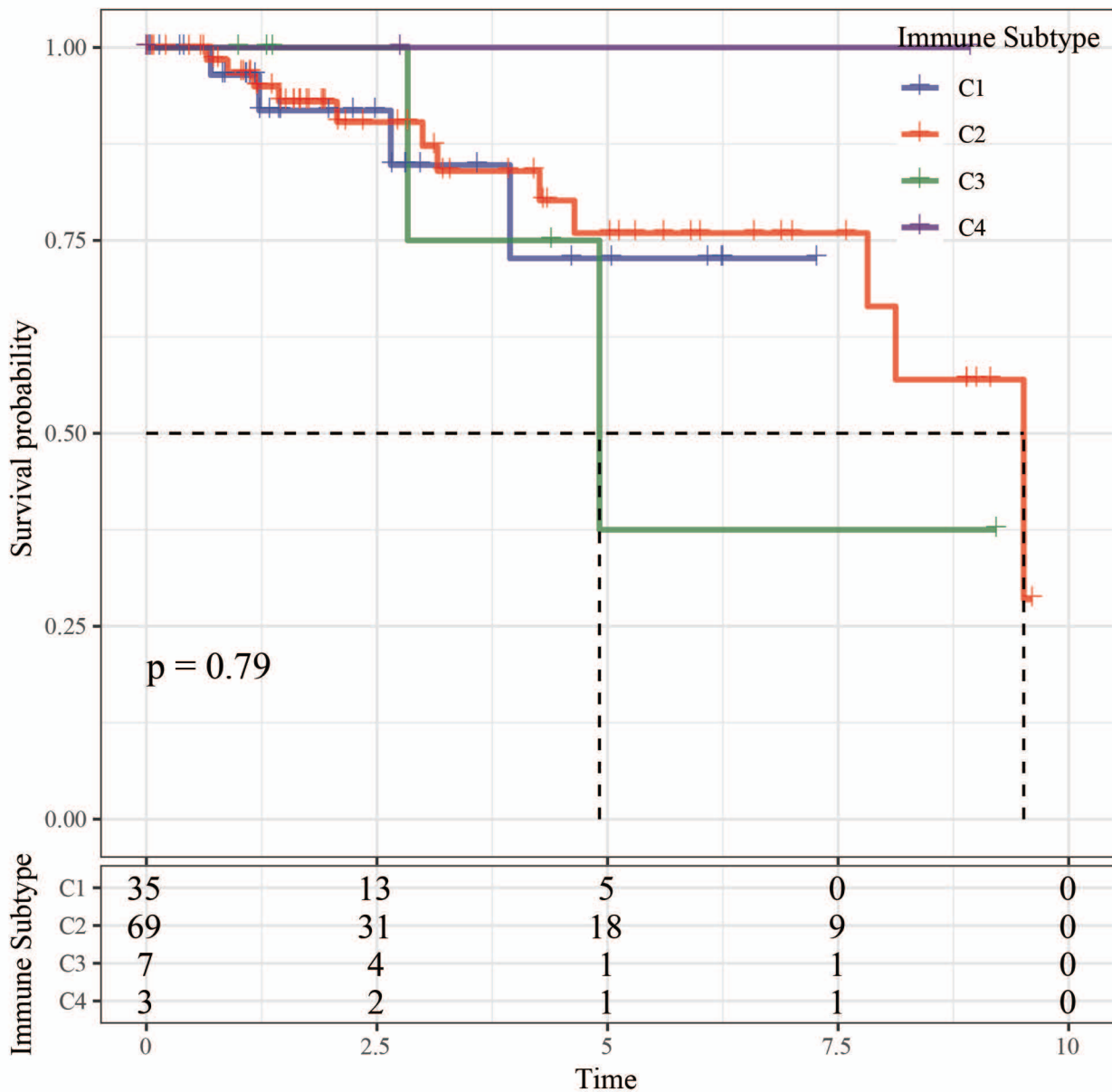

Supplement: Supplementary Figure 2 — The survival status of immune subtypes. [file Image_2.pdf]

category MC1 MC2 MC3

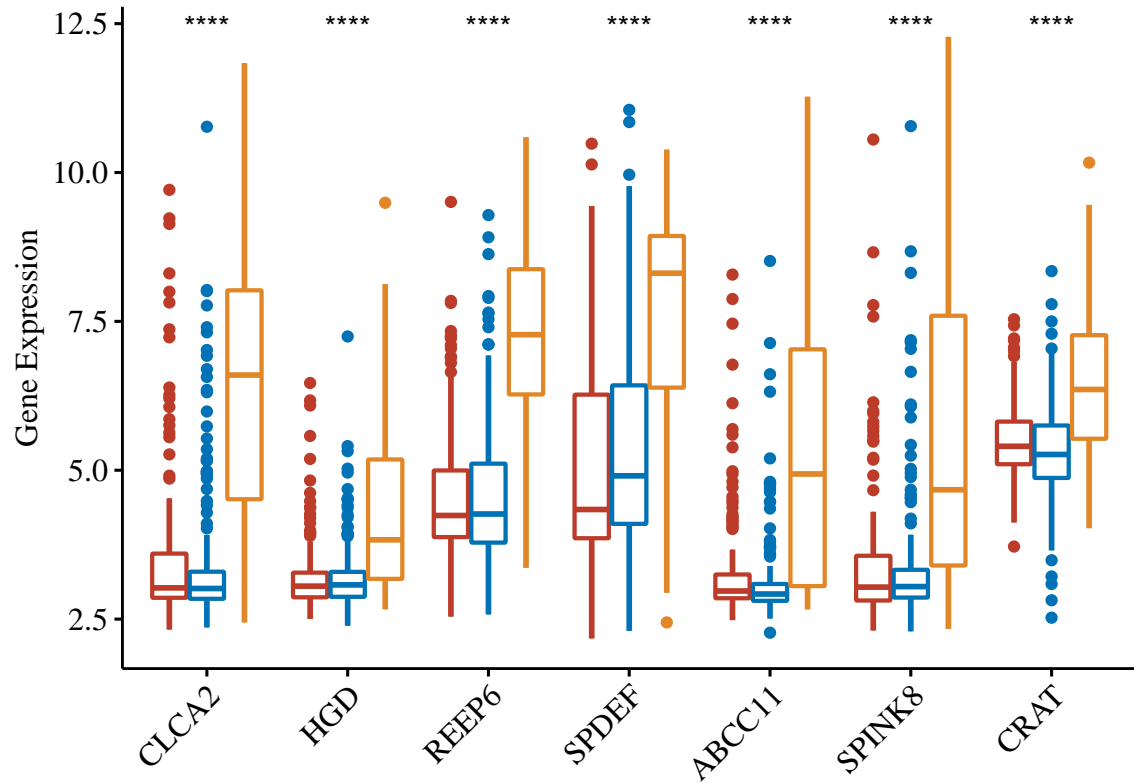

Supplement: Supplementary Figure 3 — The expression of seven key genes in three subtypes in the RNASeq dataset. Kruskal-Wallis test was performed. ****P < 0.0001. [file Image_3.pdf]

CLCA2

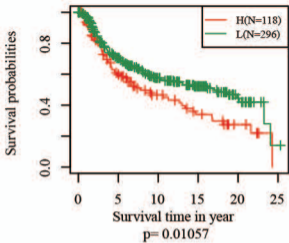

HGD

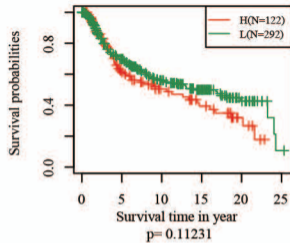

REEP6

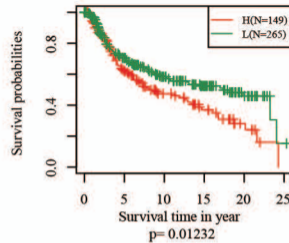

SPDEF

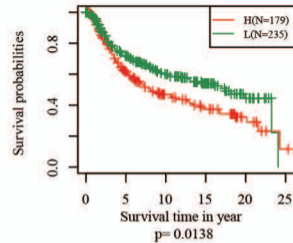

ABCC11

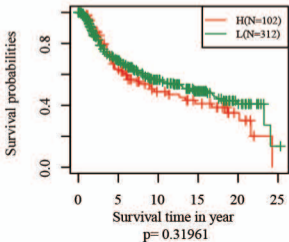

SPINK8

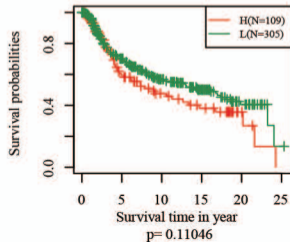

CRAT

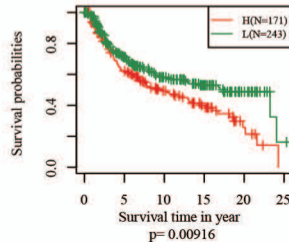

Supplement: Supplementary Figure 5 — Four genes with survival differences. [file Image_5.pdf]

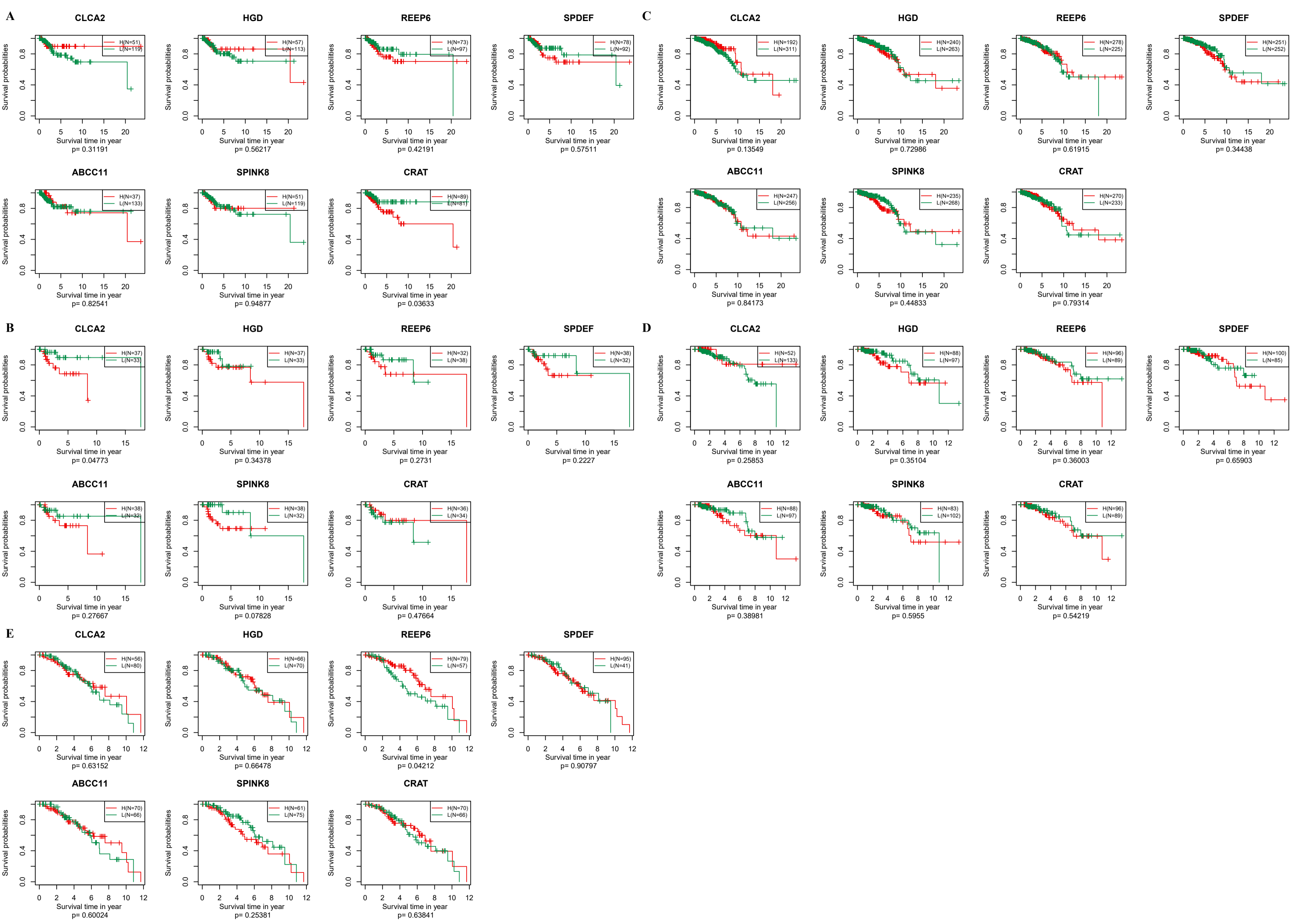

Supplement: Supplementary Figure 6 — Kaplan-Meier survival curves of five subtypes of breast cancer including basal (A), her2 (B), luminal A (C), luminal B (D), and normal (E) grouping by high and low expression groups of the seven key genes. [file Image_6.pdf]
