# Supplementary material for: Triple-Negative Breast Cancer Analysis Based on Metabolic Gene Classification and Immunotherapy
Source: Front Public Health. 2022 Jul 6;10:902378. doi: 10.3389/fpubh.2022.902378 (PMC9296841; doi:10.3389/fpubh.2022.902378)
Supplement: Supplementary Figure 4 — The PPI results of five genes (CLCA2 and SPINK8 were not shown as no interaction networks were found). [file Image_4.pdf]

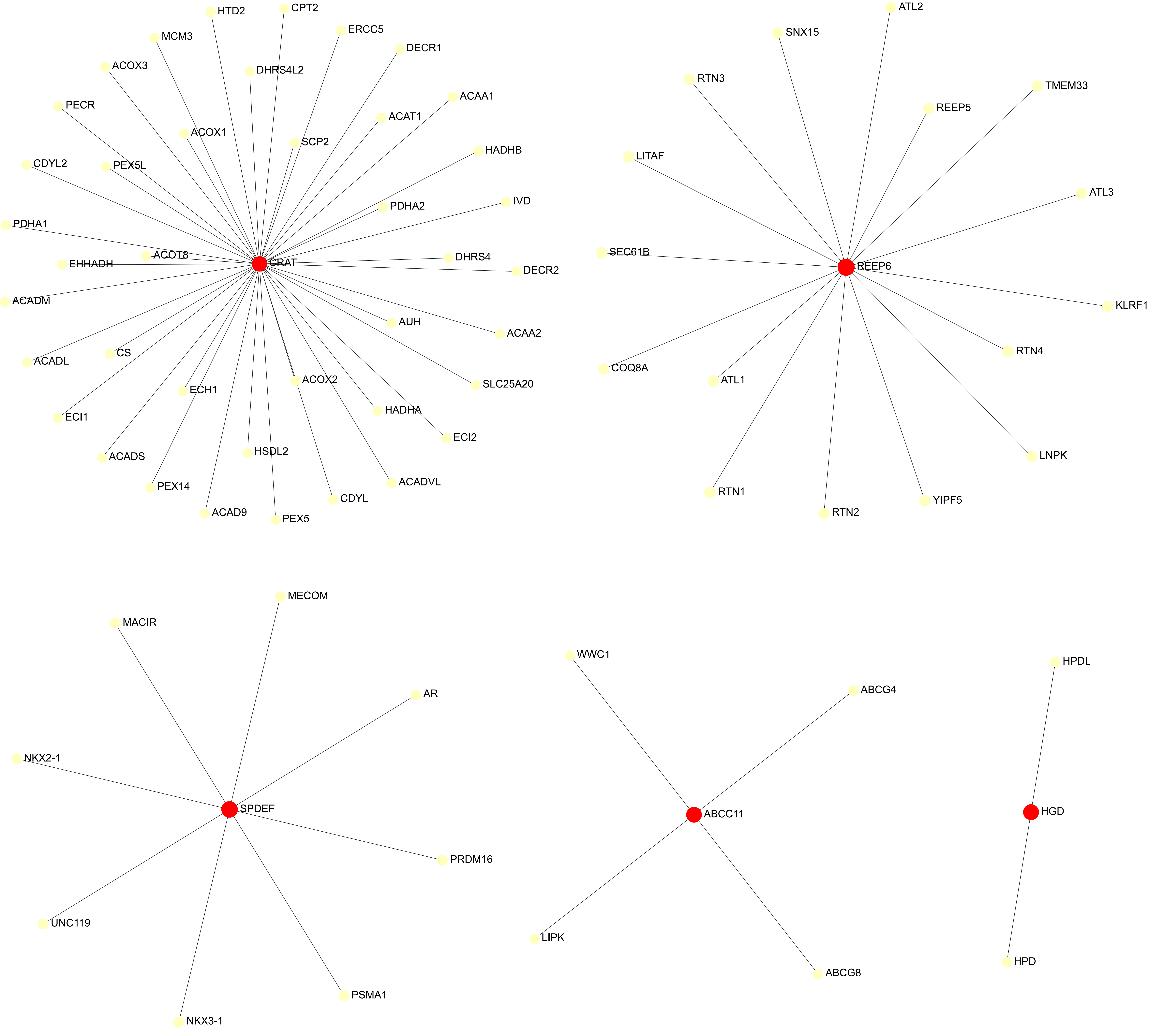

CRAT
HTD2
CPT2
ERCC5
MCM3
DECR1
ACOX3
DHRS4L2
ACAA1
PECR
ACAT1
ACOX1
SCP2
HADHB
CDYL2
PEX5L
IVD
PDHA2
PDHA1
ACOT8
DHRS4
EHHADH
DECR2
ACADM
AUH
ACAA2
CS
ACADL
ACOX2
SLC25A20
ECH1
ECI1
HADHA
ECI2
ACADS
HSDL2
PEX14
ACADVL
CDYL
ACAD9
PEX5
REEP6
ATL2
SNX15
RTN3
TMEM33
REEP5
LITAF
ATL3
SEC61B
KLRF1
RTN4
COQ8A
ATL1
LNPK
RTN1
YIPF5
RTN2
SPDEF
MECOM
MACIR
AR
NKX2-1
PRDM16
UNC119
PSMA1
NKX3-1
ABCC11
WWC1
ABCG4
LIPK
ABCG8
HGD
HPDL
HPD
